# Influenza epidemics, seasonality, and the effects of cold weather on cardiac mortality

**DOI:** 10.1186/1476-069X-11-74

**Published:** 2012-10-01

**Authors:** Stephanie von Klot, Antonella Zanobetti, Joel Schwartz

**Affiliations:** 1 Department of Environmental Health, Exposure Epidemiology and Risk Program, Harvard School of Public Health, Boston, MA, USA; 2 Helmholtz Zentrum München, German Research Center for Environmental Health, Institute of Epidemiology, Neuherberg, Germany

## Abstract

**Background:**

More people die in the winter from cardiac disease, and there are competing hypotheses to explain this. The authors conducted a study in 48 US cities to determine how much of the seasonal pattern in cardiac deaths could be explained by influenza epidemics, whether that allowed a more parsimonious control for season than traditional spline models, and whether such control changed the short term association with temperature.

**Methods:**

The authors obtained counts of daily cardiac deaths and of emergency hospital admissions of the elderly for influenza during 1992–2000. Quasi-Poisson regression models were conducted estimating the association between daily cardiac mortality, and temperature.

**Results:**

Controlling for influenza admissions provided a more parsimonious model with better Generalized Cross-Validation, lower residual serial correlation, and better captured Winter peaks. The temperature-response function was not greatly affected by adjusting for influenza. The pooled estimated increase in risk for a temperature decrease from 0 to −5°C was 1.6% (95% confidence interval (CI) 1.1-2.1%). Influenza accounted for 2.3% of cardiac deaths over this period.

**Conclusions:**

The results suggest that including epidemic data explained most of the irregular seasonal pattern (about 18% of the total seasonal variation), allowing more parsimonious models than when adjusting for seasonality only with smooth functions of time. The effect of cold temperature is not confounded by epidemics.

## Background

**Figure 1:**
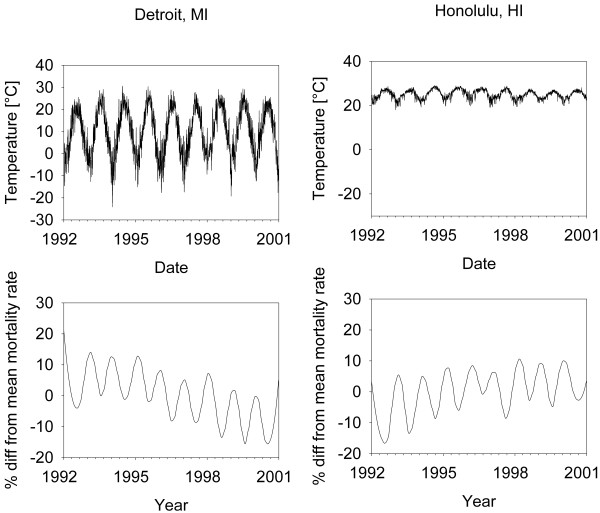
Daily Temperature in Detroit and Honolulu (upper Panel), and smoothed percent Difference from the mean Mortality Rate in each City (lower Panel).

Epidemiological studies have shown that extremes in ambient temperature are associated with short term increases in mortality [1-4]. While cold weather is associated with increased winter time deaths, it is unlikely to explain the seasonal pattern of rising and falling mortality rates. To see this, consider Figure 1 (upper panel), which shows the daily temperature vs. time in Detroit and Honolulu, and Figure 1 (lower panel), which shows a smoothed plot of the percent difference from the mean mortality rate in each city. There are similar patterns of winter time increase in both cities despite Honolulu having both an order of magnitude lower oscillation in temperature and much higher winter time temperatures than Detroit.

Therefore, most time series studies of the effects of temperature or air pollution have used functions of time, such as trigonometric functions [5], natural splines [6-9] and smoothing functions [2,10-12] to capture the potential confounding by the unnamed confounders that produce the seasonal pattern. These flexible approaches showed that seasonal patterns varied widely from year to year in the height and shape of the winter time peaks in cardiac deaths. Since the confounders are unnamed and controlled by capturing seasonal scale temporal fluctuations with functions of time, there can be much debate about the number of degrees of freedom necessary to adequately control for confounding.

Recently, Reichert and coworkers have hypothesized that the principle reason for the winter time increase in cardiac deaths is respiratory, primarily influenza epidemics [13]. Using monthly, nationwide data, they showed that influenza pandemics were associated with a downward shift in the age of the cardiac deaths, similar to the shift in severe influenza cases, and that year to year variations in the timing of the peaks of the influenza epidemic coincide with year to year shifts in the timing of the peak of cardiac deaths. Since an influenza virus infection may cause inflammatory processes which are accompanied by large increases and C reactive protein [14,15], this pattern has some biological plausibility.

This raises the following questions with respect to modeling the temporal pattern of cardiac mortality in time series studies: can we better adjust for confounding with a more parsimonious model using epidemics of influenza rather than arbitrary functions of time? How much of the seasonal pattern of the cardiac mortality can be explained by influenza epidemics? And, does control for respiratory epidemics change the observed association between cold weather and increased cardiac death?

To examine these questions, we conducted a large multi city study in the US evaluating whether adjustment for the counts of influenza admissions in the same city changes the exposure response function of temperature, whether it improves the overall model fit and whether controlling for it the remaining seasonal pattern could be modeled more simply.

## Methods

### Data

#### Mortality data

Daily mortality data from 48 cities in the US (all ages) was obtained from the National Center for Health Statistics for the time period from 1992 to 2000. The data included individual information on primary and secondary causes of death and other personal characteristics. We included cardiac causes of death (ICD 9 390–429, ICD 10 I01-I51) in the analyses. We chose cities to represent the range of weather patterns seen in the U.S.

#### Influenza data

Hospital admissions of persons age 65 years and older in the above cities were extracted from Medicare files obtained from the Health Care Financing Administration for the years 1992–2000. We calculated city specific daily counts of urgent and emergency hospital admissions with primary or secondary causes of influenza (ICD 9 487) in the 48 cities and their adjacent counties.

#### Meteorology data

For each city, we obtained hourly weather data from the nearest National Weather Service Surface Station (Earthinfo Inc, Boulder, CO, USA). We calculated daily mean values of relative humidity, air pressure and temperature.

### Analytical methods

#### Poisson regression models

We conducted separate Quasi-Poisson regression models to estimate the association of daily cardiac mortality and ambient temperature in each of the 48 US cities.

All models included cubic regression splines of same day relative humidity and air pressure with two degrees of freedom each and of temperature with four degrees of freedom, as well as day of the week as categorical variable. The base model (model 1) included a cubic regression spline of date with five degrees of freedom per year (i.e. 45 degrees of freedom for 9 years of data) to capture trend and seasonality (Table 1). This reflects the standard approach in air pollution epidemiology and studies of the impact of cold and hot days on mortality.

**Table 1:** Covariates included additionally to smooth Functions of Temperature, relative Humidity and Air Pressure, and 6 Terms for Weekday in Quasi-Poisson Regression Models 1 to 5, and total Degrees of Freedom

**Model**	**Effective degrees of freedom**	**Degrees of freedom of smooth function of time**	**Smooth function of influenza admissions, mean of lags 0–4, df = 4**	**Sinusoidal function of time with period of 1 year**
Main analyses				
1	60	5 per year (45)		
2	31	10	x	x
Sensitivity Analyses				
1’	64	5 per year (45)	x	
2’	27	10		x

The more parsimonious alternative model (model 2) uses a regression spline of a five day running mean of influenza hospital admissions (mean of same day and the previous four days) with four degrees of freedom to capture the irregular component of seasonality, and sine and cosine terms with a 1 year period each to adjust for regular seasonality. In addition we included a 10 degrees of freedom regression spline of date to adjust for long-term trends. This model used half the number of degrees of freedom compared to model 1.

#### Model comparisons

To quantify the model fit we used the Generalized Cross Validation (GCV) [16] score, a measure of goodness of fit that takes into account the effective degrees of freedom of the model. The smaller the GCV score the better the fit. We examined the distributions of pairwise differences of the GCV scores of two models. Positive values indicate a better fit of the alternative model than the base model. We also compared the partial autocorrelation functions between models to detect potential overfitting (which is suggestive when there is mostly negative autocorrelation), as well as remaining positive autocorrelation (which would be suggestive for autoregressive processes that would have to be accounted for or for underfitting). We further tested the adequacy of model choice by plotting the residuals against the linear predictors. Furthermore we plotted the observed as well as the predicted numbers of cardiac deaths versus date to evaluate how well the seasonal peaks were fit. Finally we compared the functional forms of temperature of the models for each city.

#### Summary estimate for hot and cold days

Relative risks and confidence intervals were calculated for each city specific increments in temperature using the point estimates on the exposure response function and taking into account the variance covariance matrices of estimates, following Wood 2006 [16]. Increments considered were a decrease from 0 to −5°C and from the 10^th^ to the 1^st^ percentile respectively in 24 h air temperature for cold effects and for an increase from 20 to 25°C and from the 90^th^ to the 99^th^ percentile for the heat effect. Summary estimates were obtained by pooling the city specific relative risks using a restricted maximum likelihood random effects model [17].

#### Sensitivity analyses

We added the influenza term as in model 2 to model 1 to evaluate whether more degrees of freedom are needed (model 1’). Alternatively, we excluded the influenza term from model 2 to explore the influence of influenza on the regular seasonal pattern of cardiac deaths (by comparison with model 2 (Table 1). To verify whether seasonality is properly controlled for we examined smoothed spectral density for the residuals of both models in each city.

Instead of using a penalized spline of a five day mean of influenza admission counts we modelled each influenza epidemic separately with polynomials of the length of the episode, to allow for different strengths of each epidemic as suggested by Braga et al. [18].

We had decided a priori to use temperature at lag 0 as the main exposure because we were mainly interested in acute effects of temperature. To test the robustness of the model we compared the model fit, summary estimates and the exposure response functions of the final models with models that used longer lagged averages (2-day mean of lag 0, 1 combined with 3 day mean of lag 2–5 or lag 0 combined with the mean of lag 1 to 25).

#### Quantification of seasonal variation explained by influenza epidemics

We first compared the magnitude of the trough to peak swing of the estimated sinusoidal functions of model 2 and model 2’ to obtain an estimate of how much of that peak to trough swing was explained by influenza. We then calculated the number of cardiac deaths expected due to influenza using the estimates obtained with model 2 (as exp(η + s(influenza))-exp(η)), with η incorporating all covariates other than influenza admissions, and s(influenza) being the contribution of influenza) and compared it the number of observed cardiac deaths.

## Results

**Figure 2:**
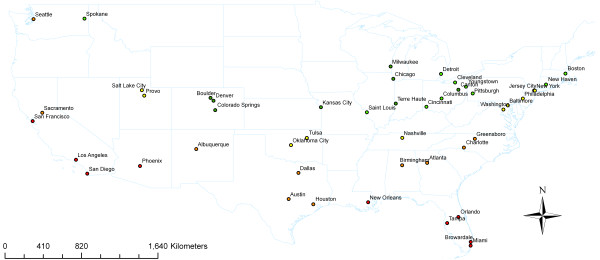
**Locations of 47 US Cities included in the Study (Honolulu not shown).** Symbol Size according to Quartiles of Population Size and Symbol Color according to Quintiles of 99^th^ Percentile of daily Minimum Temperature (green-cold to red-warm) [4].

Figure 2 shows the locations of the cities, by size and minimum temperatures for 1992 to 2000. Temperatures were lower in the north-east compared to the south-west (Table 2, Additional file 1: Figure S1). The range of temperatures differed greatly. The smallest variation of temperature was observed in Honolulu, which is not shown on the map (range: 18–29°C). Meteorology data was mostly complete with a range of 0 to 1500 missing values. The city with 1500 missing days out of 3287 war Terra Haute, where data was available only from 1996. The 25^th^ percentile of missing values was 2 for temperature and 9 for relative humidity and air pressure.

**Table 2:** Distribution of Daily mean Temperature, Influenza Hospital Admission Counts and Number of Cardiac Deaths in the Years 1992 to 2000 in the 48 US Cities included in the Study

**City, State**	**Cardiac Disease Mortality (N)**			**Influenza Hospital Admissions (N)**			**Mean Daily Temperature (°C)**		
	**Min**	**Mean**	**Max**	**Min**	**Mean**	**Max**	**Min**	**Mean**	**Max**
Albuquerque,	0	5.2	14	0	0.06	5	−9.5	14.2	32.7
Atlanta, GA	7	24.5	51	0	0.20	16	−10.7	16.9	32.1
Austin, TX	0	5.1	16	0	0.07	8	−4.0	20.4	35.2
Baltimore, MD	7	23.4	53	0	0.16	8	−17.6	13.1	32.7
Birmingham, A	1	11.6	27	0	0.19	8	−12.0	17.1	32.4
Boston, MA	0	33.2	63	0	0.55	16	−16.4	10.7	31.7
Boulder, CO	0	1.4	7	0	0.12	5	−22.4	10.0	29.4
Broward, FL	8	24.0	46	0	0.12	4	8.1	24.8	30.9
Canton, OH	0	6.2	19	0	0.16	8	−25.9	9.8	28.1
Charlotte, NC	0	7.0	20	0	0.13	7	−9.9	16.1	32.0
Chicago, IL	0	62.6	348	0	0.75	41	−26.5	10.1	32.9
Cincinatti, O	0	13.5	35	0	0.13	6	−23.5	12.1	31.4
Cleveland, OH	0	25.1	52	0	0.26	10	−23.8	10.4	29.7
Colorado Spri	0	3.2	10	0	0.12	5	−22.5	9.4	27.7
Columbus, OH	0	13.8	29	0	0.09	6	−24.3	11.7	31.6
Dallas, TX	5	18.8	38	0	0.21	16	−7.6	19.0	36.3
Denver, CO	0	6.6	17	0	0.12	5	−22.4	10.0	29.7
Detroit, MI	0	29.1	55	0	0.25	17	−24.1	10.1	30.6
Greensboro, N	0	4.8	15	0	0.11	8	−13.2	14.5	30.7
Honolulu, HI	0	9.0	26	0	0.07	3	18.0	25.0	29.1
Houston, TX	0	28.1	54	0	0.26	13	−1.2	20.5	32.7
Jersey City,	0	7.5	22	0	0.25	19	−16.3	12.9	34.2
Kansas City,	2	13.1	32	0	0.19	12	−20.8	12.3	32.2
Los Angeles,	63	103.9	206	0	0.78	30	8.3	17.2	27.5
Miami, FL	13	33.0	59	0	0.11	5	8.1	24.8	30.9
Milwaukee, WI	3	12.9	32	0	0.28	19	−26.7	9.1	33.1
Nashville,TN	0	9.2	23	0	0.17	13	−15.3	15.4	32.1
New Haven, CT	0	13.0	32	0	0.29	15	−18.2	10.2	30.9
New Orleans,	0	7.3	20	0	0.11	5	−3.3	20.6	31.3
New York City	0	142.1	261	0	0.32	18	−16.1	13.0	34.1
Oklahoma City	2	11.0	25	0	0.13	9	−14.6	15.6	34.5
Orlando, FL	0	9.3	26	0	0.18	10	2.8	22.0	31.5
Philadelphia,	0	24.7	54	0	0.22	10	−17.7	13.3	33.0
Phoenix, AZ	10	26.4	55	0	0.25	10	5.1	23.6	41.0
Pittsburgh, P	0	25.6	50	0	0.36	12	−24.4	10.9	30.0
Provo, UT	0	1.8	8	0	0.10	4	−13.6	11.8	31.9
Sacramento, C	2	13.5	31	0	0.14	9	−0.7	15.9	33.5
Salt Lake Cit	0	5.9	16	0	0.10	4	−13.6	11.8	31.9
San Diego, CA	0	28.9	60	0	0.26	13	9.6	17.4	28.2
San Francisco	0	11.6	28	0	0.22	9	3.0	14.0	28.4
Seattle, WA	4	14.9	31	0	0.18	9	−6.6	11.3	27.6
Spokane WA	0	5.0	14	0	0.13	7	−23.1	8.8	30.7
St. Louis, MO	7	23.2	44	0	0.54	41	−19.9	13.8	33.7
Tampa, FL	3	12.1	27	0	0.16	7	3.0	22.5	31.2
Terra Haute,	0	2.1	9	0	0.18	7	−20.7	11.7	30.7
Tulsa, OK	1	8.2	22	0	0.12	6	−15.7	15.8	34.7
Washington, D	0	11.0	26	0	0.13	7	−16.6	14.3	33.8
Youngstown, O	0	6.2	19	0	0.18	8	−25.2	9.5	28.9

Mean number of cardiac deaths per day ranged from 1.4 in Boulder, CO to 142 in New York City, NY, reflecting the population size of the included areas (Table 2, Additional file 2: Figure S2. The mean daily number of hospital admissions for influenza was below 1 in all cities, highest numbers of admissions on one day ranged from 3 in Honululu, HI to 41 in Chicago, Il, (Table 2, Additional file 3: Figure S3)).

**Figure 3:**
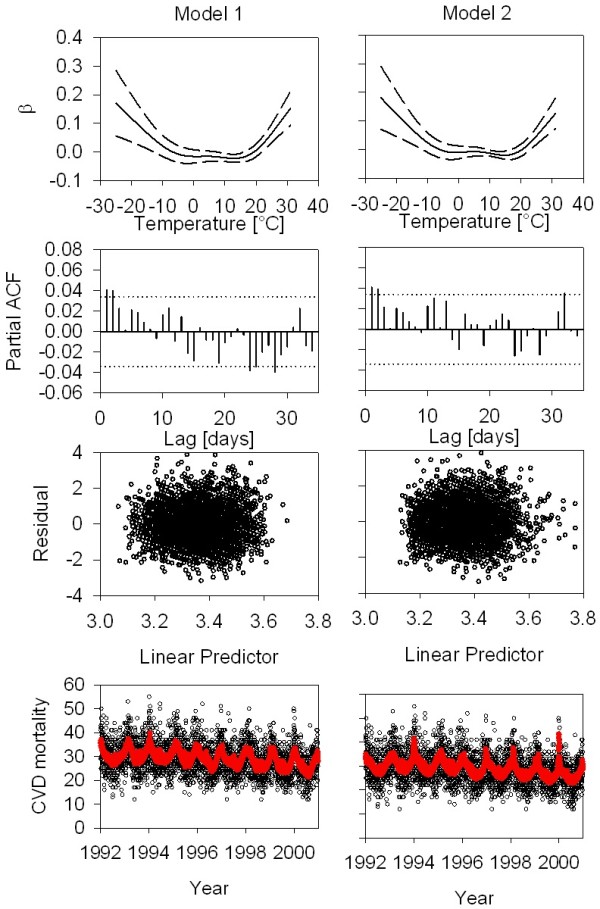
Comparison of exposure response functions, partial autocorrelation functions, residuals versus linear predictor, and observed/predicted versus time for models 1 and 2 and Detroit data.

**Figure 4:**
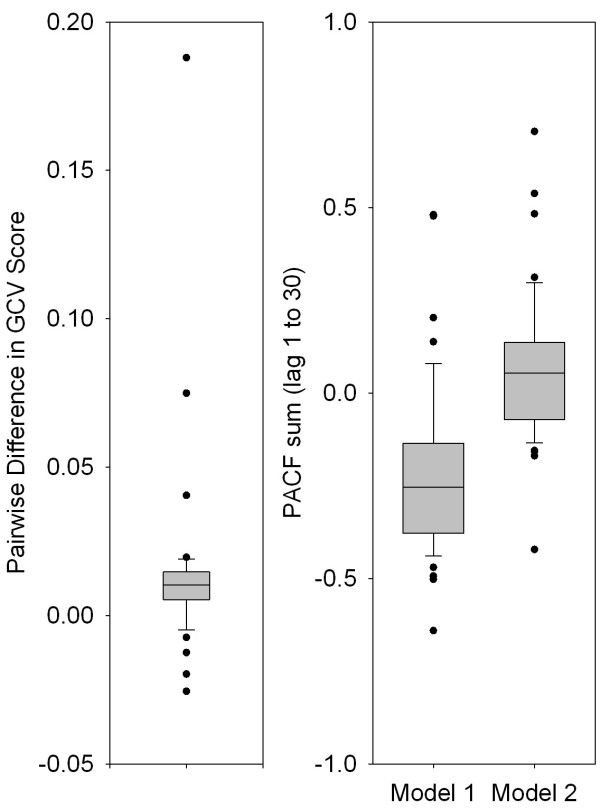
Box Plots of the 48 City-Specific Paired Differences between Generalized Cross-Validation Scores of Model 1 and Model 2 (Left Panel), and of the Sum of Partial Autocorrelation Function (PACF) of the Residuals (lag 1 to 30) (Right Panel) of Model 1 and Model 2.

Figure 3 shows plots of predicted temperature-mortality dose response curves, and diagnostics (partial autocorrelation plots, residuals, and observed and predicted values versus time) of models 1 and 2 for Detroit as an example, a city with a wide temperature range and relatively high daily event numbers. Model 1 fit the data well. The temperature response curve was U-shaped. Model 2, while using half the number of degrees of freedom compared to model 1, also fit the data well, capturing the regular seasonal pattern as well as the irregular pattern. In particular, it captured somewhat better the higher than average winter peaks in 1994 and 2000. Figure 4 (left panel) shows that, based on GCV scores across cities, model 2 fit the data better than model 1. The right panel shows the distributions of sum of the partial autocorrelation function for both models, indicating that model 1 may be somewhat overfit, whereas the mean PACF sum is 0 in model 2.

The temperature response function between daily mean temperature and mortality was mostly U or J shaped, and similar when comparing model 1 to model 2 (Figure 3 and Additional file 4: Figure S4 and Additional file 5: Figure S5). The minimum of the U-curve appeared to depend on the temperature range of the respective city. Looking at the geographical locations the U shaped curves appear more frequent in the middle areas of the country, from the west to the east coast, while J shape with a stronger cold effect occurred more in the north, with a tendency to the east, and J- shape with warm temperature effect are more frequent in the coastal south.

Estimates of the temperature cardiac mortality association were heterogeneous. Therefore pooled results were obtained under a random effects model. The overall estimated increase in risk associated with a temperature decrease from 0 to −5°C was 1.5% (95% confidence interval (CI): 1.2, 1.9%) in model 1 and 1.6% (95% CI: 1.1, 2.1%) in model 2 (Table 3). The I^2^ statistic for percent of heterogeneity was 12% for model 1, and 29% for model 2. Note that only 35 cities with a temperatures as low as −5°C could be included. The pooled relative risk (RR = for a decrease from the 10^th^ to the 1^st^ percentile, calculated to reflect region specific relative temperature decreases, was 2.7% (95%CI: 1.8, 3.6%) for model 1 and 3.1% (95%CI: 2.2, 4.0%) for model 2, with I^2^ statistics of 35% and 39% respectively.

**Table 3:** Pooled results of estimated temperature cardiac mortality association, by model (random effects model)

**Model**	**Change in temperature**			
	**0 to −5°C**	**10**^**th**^**to the 1**^**st **^**percentile**	**20 to 25°C**	**90**^**th**^**to 99**^**th **^**percentile**
	**RR (95% CI)**	**RR (95% CI)**	**RR (95% CI)**	**RR (95% CI)**
1	1.015 (1.012, 1.019)	1.027 (1.018, 1.036)	1.026 (1.019, 1.034)	1.027 (1.019, 1.036)
2	1.016 (1.011, 1.021)	1.031 (1.022, 1.040)	1.024 (1.017, 1.031)	1.030 (1.024, 1.036)

The pooled relative risk for an increase in air temperature from 20 to 25°C was 2.6% (95% confidence interval (CI): 1.9, 3.4%) in the base and 2.4% (95% CI: 1.7, 3.1%) in the alternative model, with I^2^ statistics of 60% and 67%. The pooled RR for an increase from the 90^th^ to the 99^th^ percentile was 2.7% (95%CI: 1.9, 3.6%) for the base and 3.0% (95%CI: 2.4, 3.6%) for the alternative model, with I^2^ statistics of 56% and 55%. Hence control for influenza epidemics did not diminish the effect of temperature, and heterogeneity of the temperature effects was greater for the effects of heat than the effects of cold.

Sensitivity analyses that added influenza admissions to model 1 (model 1’) resulted in GCV scores similar to model 1, some overfit of the data and no change in shape of temperature mortality functions. Model 2’, which eliminated the influenza term from model 2, clearly underfit the data.

When we characterized epidemics based on the percentiles or allowed for different effects of each epidemic the model fit was not improved over model 2 and the exposure response functions for temperature were not altered substantially. Furthermore, including hospital admissions for influenza this way, more degrees of freedom were needed than in our approach. Therefore we concluded that including a smooth function of the count of admissions allows adequate control for influenza epidemics.

When we compared our final models with models that used longer lagged averages (2-day mean of lag 0, 1 combined with 3 day mean of lag 2–5 or lag 0 combined with the mean of lag 1 to 25) there was no improvement of the model fit. The exposure response functions for the mean of lag 0 and 1 did not differ greatly from the result for the same day average. The summary estimate for a decrease from 0 to −5°C of the average of lag 0 and 1 temperature was marginally higher, but using that metric the conclusions of this investigation would not be altered. It is important to note that a moving average of 25 days of temperature may reflect seasonality more than the temperature effect.

### Seasonal variation explained by influenza epidemics

When comparing the model 2’ that did not include influenza admissions and model 2 that did include this term, on average about 18% of the peak to trough swing were explained by influenza with a range from 4% to 34%.

The estimate of influenza related cardiac deaths in each city over the study period added up to 69,714 over the cities, 2.3% of all cardiac deaths observed.

## Discussion

In the present large multi-city study in the US we showed that the association of cardiac mortality with ambient temperature can be more parsimoniously fit by including influenza data into the time series analyses. Such models better capture Winter peaks in cardiac deaths, have better GCV scores, and, unlike the more traditional spline models, do not induce negative serial correlation. We found that a regular seasonal pattern remained that could be fit by trigonometric functions. Some remaining temporal patterns needed to be captured by splines but with many fewer degrees of freedom. The models fit better than when adjusting for seasonality with a high degrees of freedom spline of time. The use of spline of time as a covariate in time series models is based on the belief that long wavelength (e.g. seasona) variations in omitted variables are likely correlated with exposure but shorter term variations are not. The choice of the cutpoint is not determinable from the data. While using many degrees of freedom may seem like a conservative approach, that risks substantial diminution of power. For example, high degree of freedom splines routinely fit peaks during summer heat waves, which removes variations in deaths we want attributable to temperature. Our finding that using a real covariate (influenza) fits the data better with fewer degrees of freedom indicates such power loss is not necessary. Indeed, while model 2 fit the rougher winter part of the mortality curve better, it used fewer degrees of freedom to fit the summer trough in mortality, which likely explains the tighter confidence intervals for the heat effects in model 2.

Model 2 also suggests that much of the irregular seasonal pattern can be explained by influenza. We also found that on average 18% (up to 34%) of the regular seasonal pattern of cardiac deaths was attributable to influenza, confirming it is an important risk factor for cardiac deaths. The presence of a remaining regular sinusoidal component after control for influenza indicates that there is more to the seasonal pattern in cardiac deaths than influenza, and that this is a more regular phenomenon (that is, similar from year to year). Length of day, hours of sunshine, vitamin D, and other variables with more regular seasonal patterns need to be investigated to parse this pattern further.

While the models that included influenza epidemics appeared to better control for winter season increase in mortality it is important to note that the temperature mortality associations remained, and if anything the estimated effect of cold days increased slightly. Hence failure to properly control for influenza epidemics does not explain the observed increase in deaths on cold days. The associations observed in this study are in line with previous studies that showed heat [19] and cold effects of temperature [2].

We estimated that influenza related cardiac deaths over the study period added up to 69,714 over the cities, 2.3% of all cardiac deaths observed. Nevertheless the results suggest that the association of cold temperatures and mortality is not explained by an increase of epidemics of respiratory infections or a regular seasonal pattern, since the shapes of the temperature mortality function were not materially altered in those models compared to the ‘standard’ model. This is in line with a recent European multi city study that used indicators for influenza and showed consistent effects of cold weather on mortality [20]. That study did not compare their results with and without influenza as a covariate. A study on the association of air pollution and mortality included similar measures to estimate influenza as confounder [18,21].

Braga et al. [18] modeled each influenza epidemic separately with polynomials of the length of the episode, to allow for different strengths of each epidemic. When we used that approach instead of a smooth function of the number of admissions, the model fit was not improved over our final model and the exposure response functions for temperature were not altered substantially. Furthermore, including hospital admissions for influenza this way, more degrees of freedom were needed than in our approach. Therefore we concluded that including a smooth function of the count of admissions allows adequate control for influenza epidemics.

In sensitivity analyses we used pneumonia admission counts as a proxy of influenza epidemics as done in previous studies [18]. This includes pneumonia caused by pathogens other than influenza, which may also be causes for heart disease morbidity and at the same time omits influenza outbreaks that do not produce much life-threatening illness. We used this as an alternative index to the influenza admissions because it may be a better and more robust estimate for epidemics in cities without large populations, since counts of influenza admissions or sentinel physician visits are generally very low on a local scale. The results were not affected with this alternative definition.

Cardiac mortality of all ages was used in the analyses to reflect the overall pattern of occurrence. Influenza epidemics in the elderly were used as a proxy of periods of influenza epidemics in the total population assuming that temporal patterns of influenza in the elderly and younger people likely overlap.

In this study on the temperature effects on cardiac mortality we did not adjust for air pollution. The reason was that there were many cities with sparse air pollution data (i.e. PM10 and Ozone). Since previous studies have shown that confounding of the cold effect by air pollution is not very strong [6,7] we preferred to use a complete time series over adjustment for it that would have meant a substantial loss of power.

## Conclusions

In conclusion we showed in a multi-city study that the association of cardiac mortality with ambient temperature could be parsimoniously fit by including influenza data into the time series analyses, while the temperature mortality association was not substantially different from the ‘standard’, less parsimonious model.

## Abbreviations

CI: Confidence interval; RR: Relative risk; GCV: Generalized Cross Validation; PM_10_: Particulate matter with a 50% cut-off aerodynamic diameter of < =10 μm.

## Competing interests

The authors declare that they have no competing interests.

## Authors’ contributions

SvK performed the statistical analyses, and drafted the manuscript. AZ supported the statistical analyses and the drafting of the manuscript. JS conceived of the study, and participated in its design and helped to draft the manuscript. All authors read and approved the final manuscript.

## Supplementary Material

Additional file 1 **Figure S1.** Box plots of daily mean temperature in the 48 cities, 1992 to 2000. Click here for file

Additional file 2 **Figure S2.** Boxplots of daily Cardiac mortality counts in the 48 cities, 1992 to 2000. Click here for file

Additional file 3 **Figure S3.** Boxplots of daily influenza hospital admission counts in the 48 cities, including adjacent coutnies *, 1992 to 2000. Click here for file

Additional file 4 **Figure S4.** City-specific plots of the smoothing function (solid line) of temperature of model 1, with 95% CI (dashed line). Click here for file

Additional file 5 **Figure S5.** City-specific plots of the smoothing function (solid line) of temperature of model 2, with 95% CI (dashed line). Click here for file
