# Supplementary figures and images for: Influenza epidemics, seasonality, and the effects of cold weather on cardiac mortality
Source: Environ Health. 2012 Oct 1;11:74. doi: 10.1186/1476-069X-11-74 (PMC3517521; doi:10.1186/1476-069X-11-74)

Additional file 1 - Box plots of daily mean temperature in the 48 cities, 1992 to 2000.

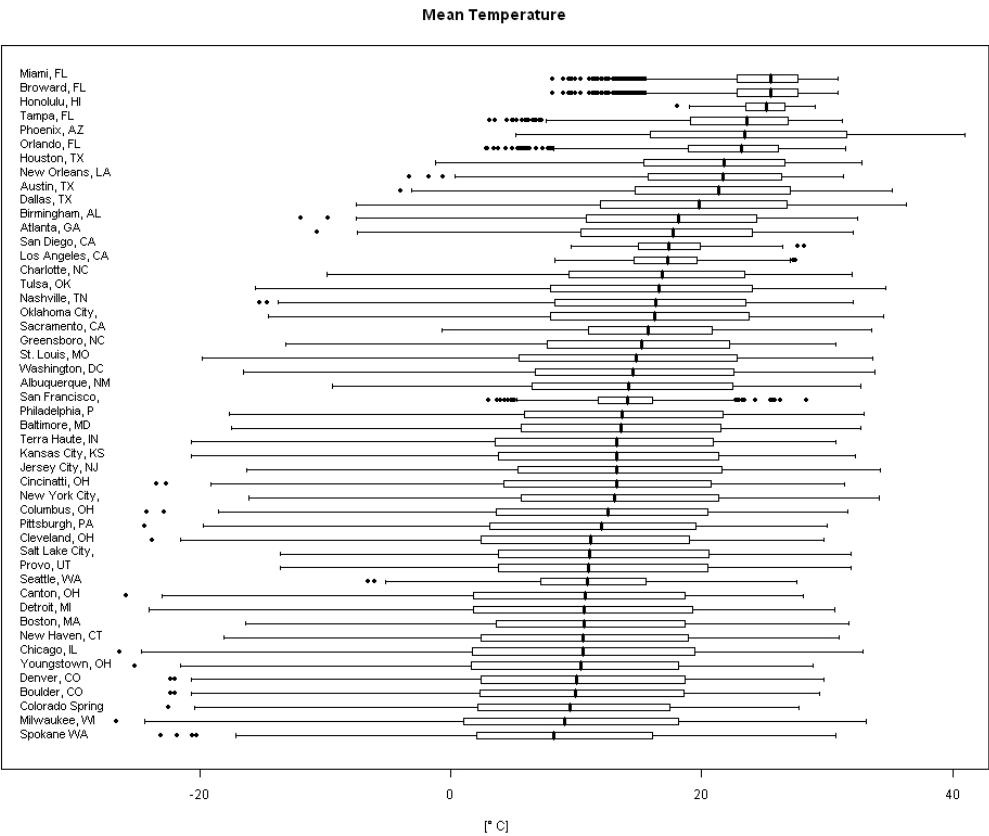

Supplement: Additional file 1 — Figure S1. Box plots of daily mean temperature in the 48 cities, 1992 to 2000. [file 1476-069X-11-74-S1.pdf]

Additional file 2 - Boxplots of daily CVD mortality counts in the 48 cities, 1992 to 2000.

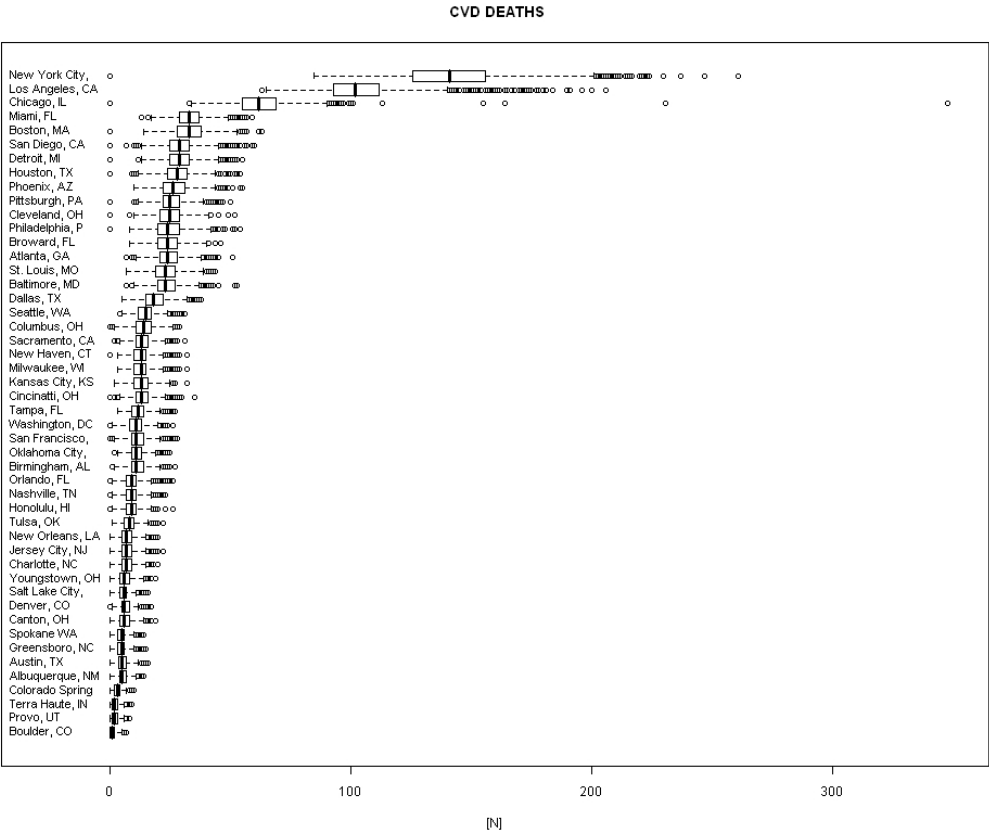

Supplement: Additional file 2 — Figure S2. Boxplots of daily Cardiac mortality counts in the 48 cities, 1992 to 2000. [file 1476-069X-11-74-S2.pdf]
