# Supplementary material for: Influenza epidemics, seasonality, and the effects of cold weather on cardiac mortality
Source: Environ Health. 2012 Oct 1;11:74. doi: 10.1186/1476-069X-11-74 (PMC3517521; doi:10.1186/1476-069X-11-74)

Additional file 3 - Boxplots of daily influenza hospital admission counts in the 48 cities, including adjacent coutnies *, 1992 to 2000.

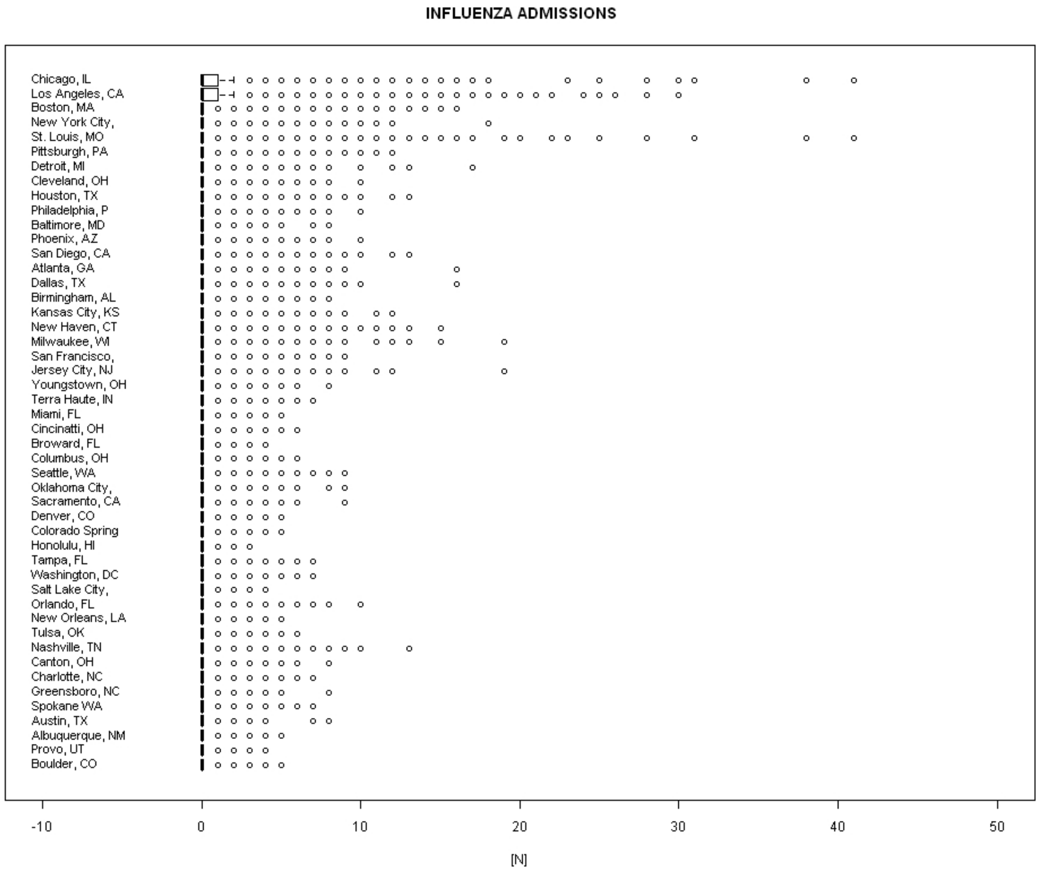

Supplement: Additional file 3 — Figure S3. Boxplots of daily influenza hospital admission counts in the 48 cities, including adjacent coutnies *, 1992 to 2000. [file 1476-069X-11-74-S3.pdf]
