# Supplementary material for: Influenza epidemics, seasonality, and the effects of cold weather on cardiac mortality
Source: Environ Health. 2012 Oct 1;11:74. doi: 10.1186/1476-069X-11-74 (PMC3517521; doi:10.1186/1476-069X-11-74)

Additional file 4 - City-specific plots of the smoothing function (solid line) of temperature of model 1, with 95 % CI (dashed line)..

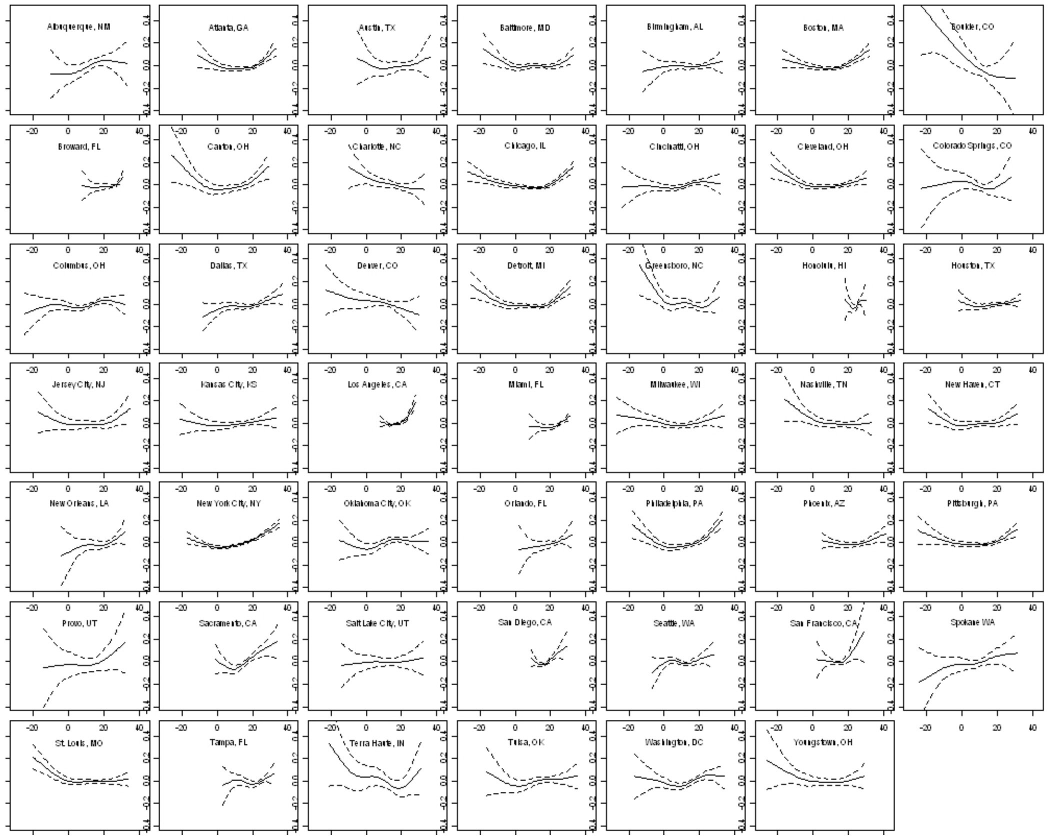

Supplement: Additional file 4 — Figure S4. City-specific plots of the smoothing function (solid line) of temperature of model 1, with 95% CI (dashed line). [file 1476-069X-11-74-S4.pdf]
